# Crystal structure of ADAMTS13 CUB domains reveals their role in global latency

**DOI:** 10.1126/sciadv.abg4403

**Published:** 2021-04-16

**Authors:** H. J. Kim, Y. Xu, A. Petri, K. Vanhoorelbeke, J. T. B. Crawley, J. Emsley

**Affiliations:** 1 Centre for Biomolecular Sciences, School of Pharmacy, University of Nottingham, Nottingham, UK.; 2 Department of Immunology and Inflammation, Imperial College London, London, UK.; 3 Laboratory for Thrombosis Research, KU Leuven Campus Kulak Kortrijk, Kortrijk, Belgium.

## Abstract

ADAMTS13 is a plasma metalloprotease that is essential for the regulation of von Willebrand factor (VWF) function, mediator of platelet recruitment to sites of blood vessel damage. ADAMTS13 function is dynamically regulated by structural changes induced by VWF binding that convert it from a latent to active conformation. ADAMTS13 global latency is manifest by the interaction of its C-terminal CUB1-2 domains with its central Spacer domain. We resolved the crystal structure of the ADAMTS13 CUB1-2 domains revealing a previously unreported configuration for the tandem CUB domains. Docking simulations between the CUB1-2 domains with the Spacer domain in combination with enzyme kinetic functional characterization of ADAMTS13 CUB domain mutants enabled the mapping of the CUB1-2 domain site that binds the Spacer domain. Together, these data reveal the molecular basis of the ADAMTS13 Spacer-CUB interaction and the control of ADAMTS13 global latency.

## Teaser

The crystal structure of the ADAMTS13 tandem CUB1 and CUB2 domains reveals how protease global latency is conferred.

## INTRODUCTION

Von Willebrand factor (VWF) is produced by both endothelial cells and platelets and secreted upon stimulation as disulfide-linked multimers containing up to ~100 VWF units (*1*). VWF functions as an essential mediator of platelet recruitment to sites of blood vessel injury (*2*). The folded conformation of circulating VWF normally conceals the binding sites for platelet glycoprotein (GP) Ibα in its A1 domain, preventing unwanted platelet binding (*2*). However, following vessel damage, plasma VWF binds via its A3 domain to exposed collagen in the subendothelial milieu. This tethered VWF then undergoes a conformational transition in response to the shear forces exerted by the flowing blood that causes exposure of the previously hidden GPIbα binding sites in its A1 domains (*2*). This process enables specific capture of platelets from blood under high shear and, in turn, facilitating platelet plug formation. As the larger VWF multimeric forms contain more collagen and platelet binding sites, and also unravel more readily in response to shear forces, they are more hemostatically competent. VWF hemostatic importance is underscored by higher plasma levels and larger multimer sizes, both increasing thrombotic risk (*3*–*5*). Conversely, low VWF levels or function are associated with elevated risk of bleeding (*6*).

VWF multimer size, and therefore its platelet tethering function, is regulated proteolytically in plasma by ADAMTS13 (*4*). ADAMTS13 is a ~190-kDa multidomain metalloprotease (MP) with an N-terminal domain organization comprising the MP, disintegrin-like (Dis), a thrombospondin type 1 (TSP) repeat, cysteine-rich (Cys-rich), and Spacer domains (*4*) these N-terminal domains are termed MDTCS. Thereafter, there are seven TSP repeats and two C-terminal CUB domains (CUB1-CUB2; hereafter termed CUB1-2) that fold back and interact noncovalently with the central Spacer domain (*7*–*10*). The importance of ADAMTS13 function is highlighted by the association of low ADAMTS13 levels with increased risk of both myocardial infarction and stroke (*3*). Severe ADAMTS13 deficiency is a hallmark of thrombotic thrombocytopenic purpura (TTP) (*4*). TTP is either inherited—caused by mutations in the ADAMTS13 gene—or more commonly acquired (i.e., through the autoimmune recognition of ADAMTS13). Anti-ADAMTS13 autoantibodies cause ADAMTS13 deficiency through enhanced antibody-mediated clearance of ADAMTS13 from plasma and also through inhibition of its enzymatic function (*11*).

There are multiple conformation-dependent exosite interactions between ADAMTS13 and VWF that determine the specificity and timing of proteolysis (*12*). Normally, VWF circulates in a globular conformation that cannot bind platelets and is also resistant to ADAMTS13 proteolysis. This is due to the fold of the central A2 domain that conceals both the ADAMTS13 cleavage site and exosite binding regions in this domain. ADAMTS13 also circulates in a latent form. ADAMTS13 latency is controlled at two levels: (i) “globally” by noncovalent interaction of the C-terminal CUB1-2 domains with the central Spacer domain that imparts a structural constraint upon the MP domain that diminishes its proteolytic function (*7*–*10*) and (ii) “locally” in the MP domain active-site itself (*13*). Local MP domain latency is stabilized by ionic interactions of the “gatekeeper triad” residues (Arg^193^, Asp^217^, and Asp^252^) that occlude the active-site cleft (*13*). This prevents off-target proteolysis by ADAMTS13 in circulation and likely also confers resistance to inhibition by plasma inhibitors, explaining the long plasma half-life of ADAMTS13 (3.5 to 8 days), which is controlled by clearance rather than inhibition (*14*).

When VWF unravels in response to elevated shear forces (e.g., at sites of vessel damage or upon secretion), it unfolds to facilitate platelet capture. The VWF A2 domain also unfolds to expose the ADAMTS13 cleavage site and exosite binding regions. ADAMTS13 first interacts with the VWF D4-CK region, which disrupts the Spacer-CUB domain–mediated “global latency” (*8*). This enhances ADAMTS13 enzyme function by about twofold by releasing some of the structural constraints on the MP domain (*15*). The Spacer and Cys-rich domains interact with the unfolded A2 domain (*16*, *17*). The Dis domain exosite then engages VWF, which transduces an allosteric change to the MP domain that unlocks the gatekeeper triad, opens the active-site cleft, and, by so doing, converts the latent form into its active conformation (*13*). This substrate-assisted allosteric activation enhances ADAMTS13 by facilitating accommodation of the scissile bond into the active-site cleft. In this unusual mode of action, VWF functions as both the activating cofactor and substrate for ADAMTS13.

How ADAMTS13 global latency is manifest remains unclear. It is apparent that global latency is stabilized by noncovalent interactions between the Spacer domain and the C-terminal CUB1-2 domains (*7*, *8*). We, and others, described a cluster of amino acids in a surface-exposed Spacer domain loop (involving Arg^660^, Tyr^661^, and Tyr^665^) that are essential for the Spacer-CUB interaction (*7*, *17*–*19*). However, the reciprocal site on the CUB1-2 domains has not been defined.

The Spacer-CUB interaction is central to ADAMTS13 global latency, and disruption of this interaction modulates ADAMTS13 function. The conformational changes induced by disruption of the Spacer-CUB interaction are of major physiological and pathophysiological relevance, as they guide enzyme function and determine its unique specificity for VWF. This conformational sensitivity also lies at the heart of the autoimmune response in acquired TTP patients, who frequently develop autoantibodies against cryptic regions in ADAMTS13 that are only exposed when ADAMTS13 opens following disruption of the Spacer-CUB interaction (*20*).

To explore ADAMTS13 global latency, we resolved the crystal structure of the ADAMTS13 CUB1-2 domains—the first structure of any C-terminal domains from an ADAMTS-family member—revealing a novel configuration for tandem CUB domains. To define the details of the ADAMTS13 intramolecular interaction, we performed docking simulations between the new CUB1-2 structure and our previously resolved crystal structure of ADAMTS13 MDTCS domains (*13*). These docking simulations, combined with targeted mutagenesis and functional characterization of ADAMTS13 variants, provide a convincing model of the intramolecular interaction site on the surface of the CUB1-2 domains, revealing how ADAMTS13 global latency is manifest at a molecular level.

## RESULTS

### Crystal structure of the ADAMTS13 CUB1-2 domains

**Fig. 1:**
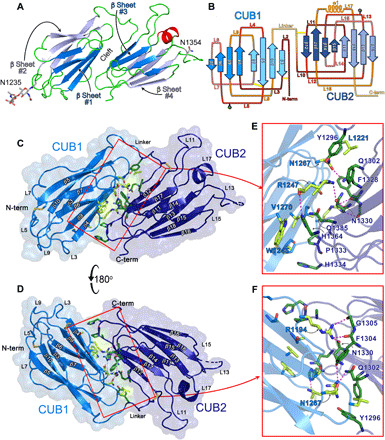
Crystal structure of the ADAMTS13 CUB1-2 domains. (**A**) Cartoon representation of the crystal structure of the ADAMTS13 CUB1-2 domains color-coded to depict secondary structures. β Strands are shown in blue, α helix is shown in red, and loops are shown in green. The β strands that form the “front” β sheets (#1 and #3) are colored in darker blue, and the β strands that form the “back” β sheets (#2 and #4) are colored in light blue. The positions of the N-linked glycans on Asn residues at positions 1235 and 1354 are labeled. The extensive interface shared by the CUB1 and CUB2 domains is denoted by the “cleft.” (**B**) Schematic representation of the CUB1-2 domain topology showing the antiparallel jelly roll fold. In each CUB domain, the darker blue β strands represent the front β sheet, and the lighter blue β strands represent the back β sheet. The location/numbering of the loops (L) and α helix as well as the locations of the N-linked glycans (green pentagon) are shown. In both CUB domains, the loops are color-coded dark red to yellow in their order from N to C terminus. The positions of disulfide bonds are shown by yellow lines. (**C** and **D**) Cartoon representations of the crystal structure of ADAMTS13 CUB1-2 in two orientations rotated forward by 180°. The β strands (β3 to β18) and the odd numbered loops (L3 to L17) are labeled. CUB1 is shown in marine blue, and CUB2 is shown in dark blue. CUB1 and CUB2 align in opposing orientations with odd numbered loops pointing in different directions. The amino acids at the CUB1-CUB2 interface are shown in stick representation. (**E** and **F**) Insets showing the amino acids involved in ionic and hydrophobic interactions that stabilize the interdomain interface.

The ADAMTS13 CUB1-2 domain expression construct spans residues Ser^1189^-Thr^1427^ with a C-terminal 6xHis tag. This was successfully expressed and secreted by Drosophila S2 cells but resulted in formation of some disulfide-linked dimers. To prevent this, we introduced the C1275S mutation to substitute the unpaired Cys^1275^. However, purification resulted in isolation of protein aggregates unsuitable for crystallization studies. We therefore fused CUB1-2 (C1275S) at its N terminus to maltose-binding protein (MBP). This was expressed in S2 cells and enabled expression of uniformly monomeric and monodisperse MBP-CUB1-2. We isolated the 70-kDa MBP-CUB1-2 fusion protein through a combination of Ni^2+^-chelating chromatography and gel filtration. Purified MBP-ADAMTS13 CUB1-2 was crystallized with the structure determined to a resolution of 2.8 Å (Fig. 1 and Table 1). The entire MBP-CUB1-2 structure is shown in fig. S1.

**Table 1:** Crystallographic data collection and refinement statistics.

**Data collection**	**MBP-CUB1-2** **PDB ID: 7B01**
Space group	*P*3_2_21
Cell dimensions	
*a*, *b*, *c* (Å)	105.6, 105.6, 125.8
α, β, γ (°)	90, 90, 120
Resolution (Å)	2.8
*R*_sym_ (%)	10.7 (134.1)
*I*/σ*I*	12.0 (1.6)
Completeness (%)	92.2 (80.0)
Redundancy	6.5 (6.0)
**Refinement**	
Resolution (Å)	2.8
No. of reflections	20,445
*R*_work_/*R*_free_ (%)	20.1/29.4
No. of atoms	4,578
Protein	4,501
Ligand/ion	51
Water	26
*B*-factors (Å)	
Protein	64.1
Metal	77.6
Water	31.6
RMSDs	
Bond lengths (Å)	0.005
Bond angles (°)	1.505

The CUB1 and CUB2 domains both exhibit a jelly roll fold with antiparallel stranded β sheets typical of CUB domains (Fig. 1, A to C) (*21*). The numbering of the β strands and loops is based on the nomenclature of other mammalian tandem CUB domains, and the four β sheets are numbered #1 to #4 from N to C terminus (Fig. 1, A to D) (*22*). A disulfide bond covalently links CUB1 loops L2 with L3 (Cys^1192^-Cys^1213^) and CUB2 loops L10 with L11 (Cys^1299^-Cys^1325^) (Fig. 1, B to D). The second disulfide bond in CUB1 (Cys^1236^-Cys^1254^) linking loops L5 and L7 is conserved between other CUB domains but missing in CUB2 (Fig. 1, B to D, and fig. S2).

In the CUB1-2 structure, the two N-linked glycans are positioned radially on Asn^1235^ and Asn^1354^, respectively, in homologous positions on loops L5 and L13 (Fig. 1, A and B). For CUB1, electron density was observed for two glycan residues attached to Asn^1235^, whereas in CUB2 the glycan attached to Asn^1354^ and part of the L13 loop are not fully resolved in the electron density (shown as a dashed segment of the cartoon representation) and are both assumed to be flexible. CUB1 and CUB2 are connected by a short linker loop (Leu^1290^-Glu^1298^) with the domains aligning in opposing orientations related by a ~180° rotation of the jelly roll structure. CUB1 β sheet #1 and CUB2 β sheet #3 both present a concave shape packing against each other at an angle of approximately 90° creating an extended axial surface, while β sheet #2 and #4 form convex surfaces located radially (Fig. 1, A, C, and D). The central interface involves six hydrogen bonding interactions between Arg^1247^-Asn^1330^, Asn^1267^-Gln^1302^, Arg^1194^-Phe^1304^, Arg^1194^-Gly^1305^, and Arg^1194^-Asn^1330^ and a network of hydrophobic contacts (Fig. 1, E and F). On the axis of the CUB1-2 interface, the His^1364^ side chain from CUB1 loop L6 forms a hydrogen bond with the main chain of Arg^1247^ from the CUB2 L14 loop.

**Fig. 2:**
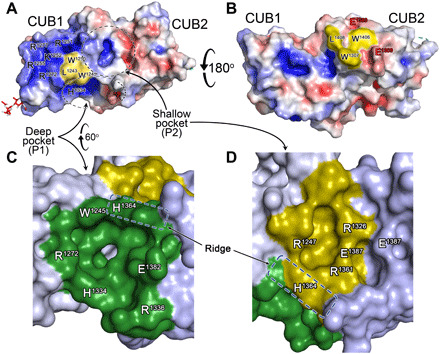
Surface features of the ADAMTS13 CUB1-2 domains. (**A** and **B**) Surface representation of the ADAMTS13 CUB1-2 domains, CUB1 (left) and CUB2 (right), are labeled. Positive surface charge is shown in blue, and negative charge is shown in red. CUB1-2 domains are presented in two orientations rotated forward by 180°. There are two hydrophobic patches [one in each CUB domain consisting of a Leu residue and two Trp residues (yellow), surrounded by labeled charged residues]. In (A), there are two discernible pockets that are circled and enlarged in (C) and (D). (**C** and **D**) The deep pocket (termed P1) shown in dark green has charged amino acids that form the rim of the pocket. The shallow pocket (P2) is shown in olive green, which is again flanked by charged residues. His^1364^ forms part of a ridge that separates the P1 and P2 pockets and is highlighted in both representations of the pockets.

The tandem CUB1-2 structure we observe is novel. The CUB1-2 domains share a much larger interface (758 Å^2^) with each other than other tandem CUB domains. CUB1-2 also has additional interesting surface features, including two hydrophobic patches that are composed of Leu-Trp-Trp residues in a triangular arrangement (Leu^1243^-Trp^1245^-Trp^1250^ in β sheet #1; Trp^1307^-Leu^1408^-Trp^1406^ in β sheet #4). These hydrophobic patches are surrounded by charged surface residues—positively charged in CUB1 β sheet #1 (Fig. 2A) and negatively charged residues in CUB2 β sheet #4 (Fig. 2B).

On either side of the interdomain cleft are two pockets: (i) a deep pocket (P1) composed of residues from CUB1 β sheet #1 and CUB2 loops L12, L14, and L16, and (ii) a shallow pocket (P2) composed of side chains from the linker loop, CUB2 β sheet #3 and CUB1 loop L6 (Fig. 2, A, C, and D, and movie S1).

### Distinctive features of the ADAMTS13 CUB1-2 structure

CUB domains exhibit a characteristic β sandwich structure composed of ~110 amino acids and are often repeated in multidomain proteins (fig. S2) (*23*). Many proteins contain tandem CUB domains (*24*). ADAMTS13, similar to neuropilin (NRP)-1, NRP-2, and procollagen C-endopeptidase enhancer 1 (PCPE-1), has a short linker that connects CUB1 and CUB2 (*25*–*28*). Other tandem CUB domain proteins like mannan-binding lectin serine protease (MASP-1) and complement proteins, C1r and C1s, have an intervening epidermal growth factor (EGF)–like domain. BMP-1, like ADAMTS13, has an N-terminal MP domain. In BMP-1, the C-terminally located CUB domains are important both for substrate recognition and for controlling/restricting its proteolytic activity, which bears functional resemblance to the CUB1-2 domains of ADAMTS13 (fig. S2) (*7*, *8*, *15*, *29*, *30*).

CUB domain–containing proteins can be classified not only as either single- or multi-CUB domain but also by the presence/absence of a coordinated Ca^2+^ ion (*31*). Small single CUB domain–containing proteins, such as those of the spermadhesin family, do not coordinate Ca^2+^ in their crystal structures and also have additional β strands (β1 and β2) (*22*). The lack of β1 and β2 strands is considered a distinguishing characteristic of Ca^2+^-binding CUB domains. All the available tandem CUB domain structures coordinate Ca^2+^ ions in a conserved manner. Ca^2+^ binding can be predicted in many other multi-CUB domain proteins due to the presence of the conserved Ca^2+^-binding motif (fig. S2) (*31*). Where known, Ca^2+^ binding to CUB domains is often important for ligand/substrate binding, as well as loop stability. It is therefore notable that neither CUB1 nor CUB2 in ADAMTS13 binds Ca^2+^ or contains the conserved Ca^2+^-binding motif, despite the lack of the β1 and β2 strands (fig. S2) (*25*). Therefore, although ADAMTS13 CUB1 and CUB2 are non–Ca^2+^-binding CUB domains, the absence of the β1 and β2 strands and their tandem CUB domain organization and regulatory function make them structurally and functionally more closely linked to other tandem Ca^2+^-binding CUB domain–containing proteins.

### CUB domain comparisons

**Fig. 3:**
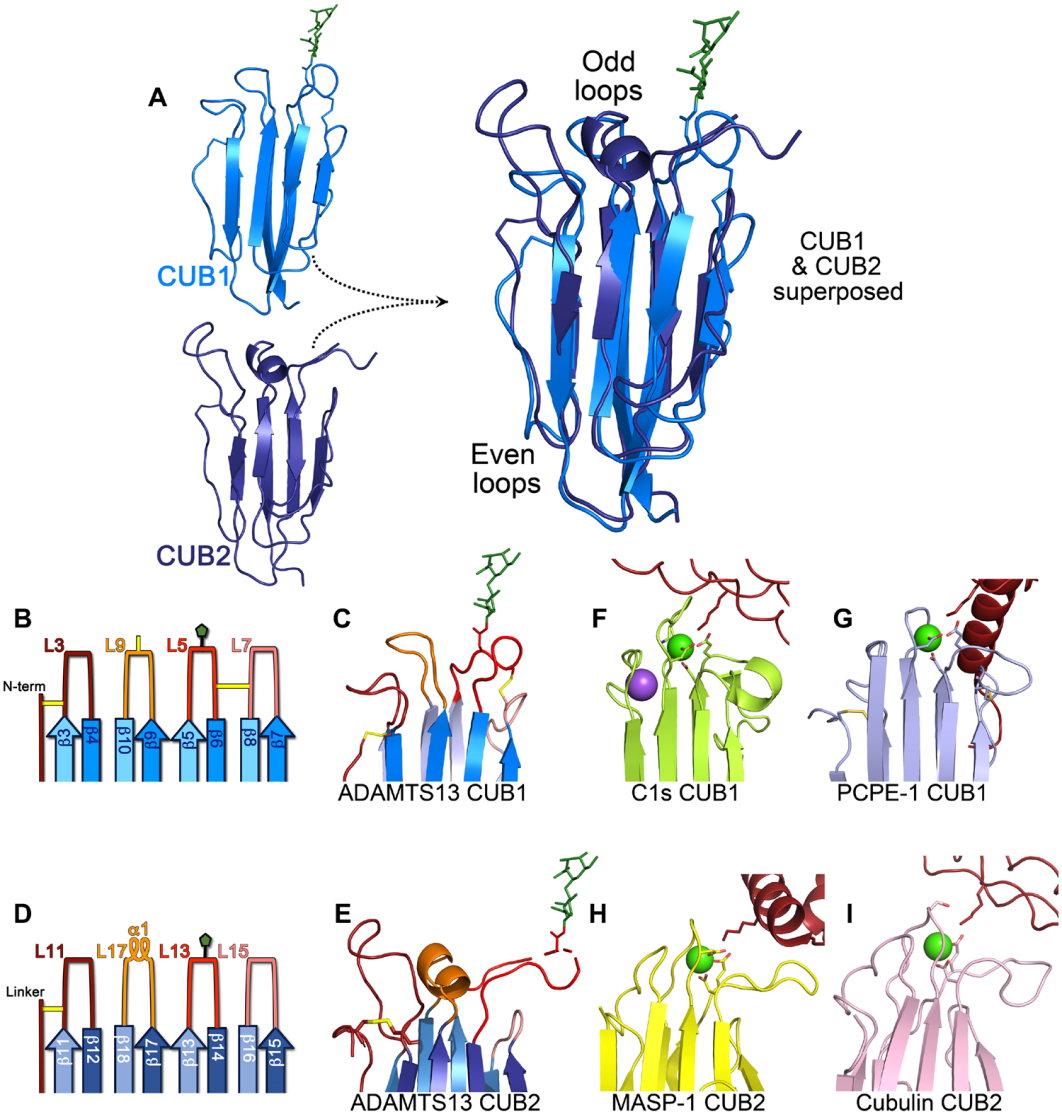
Structural features of ADAMTS13 CUB1-2 domains. (**A**) ADAMTS13 CUB1 (marine blue) and CUB2 (dark blue) domains are depicted in cartoon representation in the same orientation with the odd numbered loops pointing upward. These domains are superposed. Note the good alignment of the β strands and the even numbered loops, but appreciable differences in the odd numbered loops. (**B**) CUB1 and (**D**) CUB2 domain odd numbered loops are depicted. The front β-sheet strands are shown in darker blue, and the back β-sheet strands are shown in lighter blue. Loops (L) are labeled and color-coded from dark to light in order from N to C terminus. Disulfide bonds are shown in yellow, and the N-linked glycan is shown in green. The corresponding regions of the structures of (**C**) ADAMTS13 CUB1, (**E**) CUB2, (**F**) C1s CUB1 (PDB: 4LOR), (**G**) PCPE-1 CUB1 (PDB: 6FZV), (**H**) MASP-1 CUB2 (PDB: 3POB), and (**I**) cubulin CUB2 (PDB: 3KQ4) are shown. Note the Ca^2+^ ion (green sphere) bound by C1s and PCPE-1, MASP-1, and cubulin. For C1s and PCPE-1, MASP-1 and cubulin CUB domain ligand-bound structures are shown (ligand in red) interacting with the Ca^2+^-binding loops. C1s also binds a Na^+^ ion (purple sphere).

When CUB1 and CUB2 are superposed, their β strands and even numbered loops align well (Fig. 3A). In contrast, there is appreciable disparity in the conformation of the outward pointing, odd numbered loops. Using the CUB1 structure and searching the Protein Data Bank (PDB) for matching structures (*32*), a similar principle is observed in which the β sheets of the jelly roll fold superpose very well with other Ca^2+^-binding CUB structures. However, greater variation occurs in the odd numbered loops, which in other CUB domains contain the Ca^2+^-binding site (but not in ADAMTS13). The root mean square deviation (RMSD) values for the seven most similar CUB structures range from 2.4 to 2.9 Å, with *z* scores in the range of 8.7 to 13.0 despite amino acid identities in the range of only 13 to 18%. A similar analysis using the CUB2 structure yielded the same top seven CUB domain hits and RMSD values of 2.4 to 2.9 Å, *z* scores in the range of 8.7 to 10.7, and amino acid identities ranging from 9 to 17%. Greater disparity in structure is observed for the ADAMTS13 CUB2 structure when compared with other CUB structures. This is due to the missing disulfide, the presence of the L17 loop α helix, and extensive divergence in the odd numbered loops.

When the ADAMTS13 CUB1-2 domain amino acid sequences are aligned with other tandemly arranged mammalian CUB domain proteins, the odd numbered loops (CUB1: L3, L5, L7, and L9; CUB2: L11, L13, L15, and L17) are more divergent (fig. S2). ADAMTS13 CUB1 and CUB2 have N-linked glycosylation sites on homologous loops (L5 and L13, respectively). Glycosylation of these loops is not a conserved feature among tandemly arranged CUB domains, setting ADAMTS13 CUB domains apart (*31*).

All tandem CUB domain structures resolved to date have a conserved Ca^2+^-binding site between the two longest odd numbered loops (fig. S2). However, the negatively charged residues on loops L5/L9 (CUB1) and L13/L17 (CUB2) required for Ca^2+^ coordination are not present in ADAMTS13 and bound Ca^2+^ is not observed in the structure (Fig. 3, B to E). This is the first structure of tandemly arranged CUB domains that lack the β1 and β2 strands (a hallmark of Ca^2+^-binding CUB domains) that does not bind Ca^2+^ (*31*). From the ligand-bound structures of other tandemly arranged CUB domains, the Ca^2+^-binding loops in these CUB domains form the ligand binding site (Fig. 3, F to I), suggesting that this mode of ligand binding is not conserved in ADAMTS13 (*28*, *33*–*35*). This is further corroborated by the presence of the N-linked glycans in ADAMTS13 CUB domains that would sterically interfere with this mode of ligand binding (Fig. 3, B to E).

### Identification of the ADAMTS13 CUB1-2 site that binds the Spacer domain

The C-terminal ADAMTS13 CUB1-2 domains are involved in the intramolecular interaction with the central Spacer domain. Although the effects of this interaction in modulating both ADAMTS13 conformation and function are comparatively well understood (*7*–*10*, *15*, *36*), the molecular basis of the binding has not been characterized. The binding between CUB1-2 and the Spacer domain confers global latency to ADAMTS13, diminishing its enzymatic function. Disruption of the Spacer-CUB interaction, either physiologically when ADAMTS13 binds VWF or experimentally when the pH is dropped or with the use of certain activating monoclonal antibodies, unlocks global latency and opens up the ADAMTS13 conformation, which, in turn, enhances enzymatic function by about twofold (*7*–*10*, *15*, *36*). This conformational sensitivity of ADAMTS13 is also highly important in the autoimmune recognition of ADAMTS13 by autoantibodies in acquired TTP patients. For these reasons, understanding how the Spacer-CUB interaction is manifest is of major physiological and pathophysiological relevance.

**Fig. 4:**
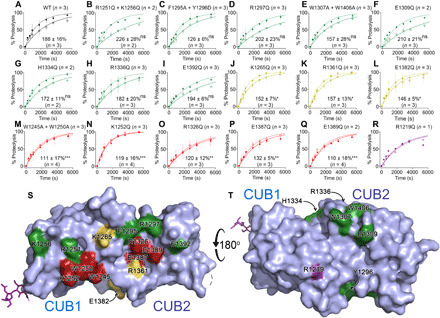
Functional analysis of full-length ADAMTS13 CUB1-2 mutants. (**A**) Recombinant full-length ADAMTS13 was analyzed functionally using VWF96 as a substrate. Proteolysis of VWF96 by 0.45 nM ADAMTS13 in the absence (solid line) and presence (dotted line) of the activating anti-CUB domain monoclonal antibody 17G2 that disrupts the Spacer-CUB interaction was quantified over time by ELISA. Progress curves are shown as mean ± SEM (*n* = 3). In the presence of 17G2, ADAMTS13 catalytic efficiency is enhanced by 188 ± 13% (mean ± SD; *n* = 4). (**B** to **R**) Parallel analysis of recombinant ADAMTS13 CUB domain mutants. The number of replicates for each dataset presented in the graph is given. The % enhancement effect of 17G2 ± SD is shown for each mutant with the number of replicates (including reactions performed at different enzyme concentrations that are not represented graphically). (B) to (I) are mutants shown in green that are activated similar to wild-type (WT) ADAMTS13. The % activation is not significantly different (ns) to WT ADAMTS13. (J) to (L) are mutants shown in yellow that exhibit partial, but significantly reduced activation in response to 17G2 (*P ≤ 0.05). (M) to (Q) are mutants shown in red that exhibit highly significantly reduced activation in response to 17G2 (**P ≤ 0.01; ***P ≤ 0.001). (R) ADAMTS13 R1219Q mutant was not activated by 17G2, but this was subsequently found to be due to the loss of binding of the 17G2 antibody. All other mutants bound 17G2 normally. (**S** and **T**) ADAMTS13 CUB1-2 domain surface amino acids and their influence upon the Spacer-CUB interaction. Amino acids depicted in fig. S4 are color-coded according to their influence on the Spacer-CUB interaction. Amino acids shown in green do not influence the Spacer-CUB interaction, those in yellow have a moderate or indirect effect, whereas those residues highlighted in red that form two adjacent patches in CUB1 and CUB2 represent amino acids that have a major influence upon Spacer-CUB binding.

Previous studies have revealed that the Spacer domain residues Arg^568^, Phe^592^, Arg^660^, Tyr^661^, and Tyr^665^ either directly contribute or are in close proximity to the surface that interacts with the CUB domains (*17*, *19*, *37*). To identify the reciprocal binding site on the CUB1-2 domains, we performed docking simulations between our CUB1-2 structure and that of previously determined crystal structures with the Spacer domain (PDB codes: 3GHM and 6QIG) using programs ClusPro and HADDOCK. Initial docking without input of suspected interfacial contact residues resulted in several orientations observed in the top 10 high scoring poses that involved the known Spacer domain exosite (the three highest scoring poses are shown in fig. S3). On the basis of these, we identified a panel of amino acids on the surface of the CUB1-2 domains that had the potential to form interactions with the Spacer domain (fig. S4). Next, we generated a panel of 17 full-length ADAMTS13 variants containing mutations in the CUB domains. All recombinant ADAMTS13 variants were secreted from human embryonic kidney (HEK) 293T cells at similar levels to wild-type ADAMTS13. To determine whether any of the mutations had disrupted the Spacer-CUB interaction, we assayed the variants kinetically using VWF96 as a specific ADAMTS13 substrate (*13*). Proteolysis of VWF96 by ADAMTS13 was performed in the presence and absence of the monoclonal anti-ADAMTS13 CUB1-2 domain antibody 17G2 (*15*). This antibody binds the CUB1-2 domains and, in so doing, unlocks ADAMTS13 global latency through disruption of the Spacer-CUB interaction (*15*). The effect of this antibody upon wild-type ADAMTS13 function was monitored kinetically, revealing a 188% increase in catalytic efficiency (Fig. 4A), similar to previous studies (*15*). We then exploited this assay to identify those ADAMTS13 variants in which the Spacer-CUB interaction was disrupted, rationalizing that those variants that could be activated by 17G2 must have an intact Spacer-CUB interaction, whereas those that could not be activated by 17G2 must already be “open”/activated. Of the 17 variants, 8 exhibited normal activation with 17G2 (Fig. 4, B to I), suggesting that the substituted residues were not of major importance in the interaction with the Spacer domain. Five variants—W1245A/W1250A, K1252Q, R1326Q, E1387Q, and E1389Q—exhibited a highly significant reduction in activation (Fig. 4, M to Q), suggesting that the substituted residues could be of particular importance in mediating the Spacer-CUB1-2 interaction. Three variants—K1265Q, R1361Q, and E1382Q—were activated by 17G2, but to a lesser extent (Fig. 4, J to L), implying that these variants may have perturbed rather than fully disrupted Spacer-CUB interaction. The last variant analyzed, R1219Q, was not activated by 17G2 (Fig. 4R). However, further analysis by enzyme-linked immunosorbent assay (ELISA) revealed that the R1219Q substitution abolished the binding of the 17G2 antibody, explaining the lack of activation of this variant. All other ADAMTS13 variants bound 17G2 normally.

### Binding interface between ADAMTS13 CUB1-2 and Spacer domains

**Fig. 5:**
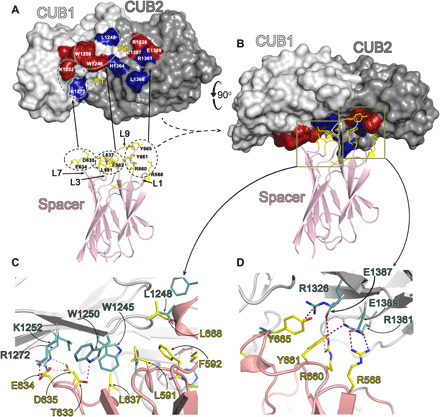
Proposed interaction between the ADAMTS13 CUB1-2 domains and the ADAMTS13 Spacer domain. (**A**) Space-fill representation of the ADAMTS13 CUB1-2 domains with residues identified from functional analyses to influence the Spacer-CUB interaction labeled in red. Those labeled in blue are amino acids predicted from the refined ClusPro docking simulation to contribute to Spacer domain binding. The positions of the P1 and P2 pockets are shown. Below is the ADAMTS13 Spacer domain that interacts with the CUB domains shown in cartoon representation. In stick representation (yellow) are the reciprocal residues that likely interact directly with the CUB1-2 domain patches. The Spacer domain loop (L) numbering for the highlighted residues is given. (**B**) Favored docking model of the CUB1-2 domains interacting with the Spacer domain. Note that the CUB1-2 domains have rolled forward by ~90° when compared to (A). Boxed areas highlight the two areas enlarged in (**C**) and (**D**), corresponding to the CUB1 and CUB2 interaction sites, respectively. (C) CUB1 interaction site with the Spacer domain. The R-groups of amino acids involved in either hydrophobic or ionic interactions are shown in stick representation and labeled (CUB residues in teal; Spacer residues in yellow). (D) CUB2 interaction site with the Spacer domain. The R-groups of amino acids involved in ionic interactions are shown in stick representation and labeled (CUB residues in teal; Spacer residues in yellow).

From the functional analyses, we identified two clusters of residues that are important for the Spacer-CUB interaction. Although these clusters are in different domains, they lie on the same contiguous surface of CUB1 and CUB2: (i) residues Trp^1245^, Trp^1250^, and Lys^1252^ locate to CUB1 β sheet #1 on the rim of the P1 pocket and (ii) Arg^1326^, Glu^1387^, and Glu^1389^ locate to CUB2 β sheet #3, near the P2 pocket (Fig. 4, S and T). In addition, the three variants (K1265Q, R1361Q, and E1382Q) that exhibited partially reduced activation by 17G2 lie in relatively close proximity to this surface (Fig. 4S). On the basis of these functional data, we performed a second round of the docking simulations using both ClusPro and HADDOCK to establish a docking pose that could uniquely pair up these residues with the Spacer domain exosite residues, Arg^568^, Phe^592^, Arg^660^, Tyr^661^, and Tyr^665^. The highest scoring pose from ClusPro had 196 members and a weighted score of −1256. The same docking pose was obtained irrespective of whether the Spacer domain coordinates from 3GHM or 6IQG were used. Reassuringly, a very similar pose was obtained independently using HADDOCK software, resulting in a score of −191. The ClusPro docking solution matched the experimental data most consistently (Fig. 4) and previous data on the involvement of specific Spacer domain residues (Fig. 5, A and B) (*7*, *19*).

The surface of the Spacer domain identified by the docking simulations to form interactions with CUB1-2 is formed by Spacer domain loops L1, L3, L7, and L9 that protrude from the end of the Spacer domain jelly roll fold (Fig. 5A and movie S2) (*38*). At the center of this surface, loop L3 forms a short hairpin loop that spans residues Asn^587^-Thr^593^ and is hydrophobic in nature with residues Leu^591^ and Phe^592^ projecting outward. Leu^637^ on the adjacent L7 loop adds to this hydrophobic patch. Also, in loop L7, which spans residues Thr^633^-Pro^638^, are the negatively charged Glu^634^ and Asp^635^. Loop L9 spans residues Arg^659^-Arg^670^ and contains Arg^660^, Tyr^661^, and Tyr^665^, all previously implicated in CUB domain binding. Adjacent to this, loop L1 spanning Thr^563^-Arg^568^ contains basic Arg^568^ next to the L9 loop.

On the basis of the highest scoring docking pose and our functional data, we propose an interaction in which Glu^634^ and Asp^635^ in the Spacer domain form salt bridges with Lys^1252^ and Arg^1272^ in the CUB1 domain (Fig. 5C). At the center of the interface, the hydrophobic patch consisting of Leu^591^ and Phe^592^ (both L3), Leu^637^ (L7), and Leu^668^ (L9) in the Spacer domain interacts hydrophobically with Trp^1245^, Leu^1248^, and Trp^1250^ in CUB1. Last, we propose that the Spacer domain loop, L9, spanning Arg^660^-Tyr^665^ is accommodated ionically by the pocket formed by Arg^1326^, Arg^1361^, Glu^1387^, and Glu^1389^ in CUB2, with Tyr^665^ side chain predicted to make a cation-Pi interaction with Arg^1326^ (Fig. 5D). There is a further contribution of Arg^568^ (L1) in the Spacer domain forming a salt bridge with Glu^1389^ in the CUB2 domain.

## DISCUSSION

The 19 ADAMTS family proteases all have the same conserved MDTCS domain organization at their N termini. Thereafter, the number and identity of the C-terminal domains vary widely according to family member and functional subgroup (*39*). ADAMTS13 is the only ADAMTS family member that contains CUB domains and also appears to be functionally and evolutionarily distinct from the other family members. In general terms, very little is known about the functional contributions of the C-terminal domains of ADAMTS family members. For ADAMTS13, recent data have suggested an important role of the C-terminal domains in modulating the proteolytic function of the N-terminal MP domain (*7*, *8*, *15*, *36*). For this, the CUB domains interact with the central Spacer domain. This is enabled by the highly flexible intervening TSP2-8 repeats that allow the CUB1-2 domains to fold back on the rest of the molecule (fig. S5). To gain insight into the function of the ADAMTS13 CUB1-2 domains, we resolved the crystal structure of the ADAMTS13 CUB1-2 domains. Purification of soluble, monodisperse CUB1-2 was only enabled through N-terminal fusion with MBP and by introduction of the C1275S mutation.

Many different and diverse proteins contain tandem CUB domains. Although some contain an intervening EGF-like domain that spatially separates CUB1 and CUB2, others including ADAMTS13 are consecutive, joined by a short linker (fig. S2). Both CUB1 and CUB2 have the characteristic jelly roll fold of Ca^2+^-binding CUB domains, consisting of two four-stranded β sheets (Fig. 1) (*31*). Although typical of the tandem Ca^2+^-binding CUB domains, neither the ADAMTS13 CUB1 nor CUB2 binds Ca^2+^ (Figs. 1 and 3). For those consecutive tandem CUB domains for which there are structural data, the interface between CUB1 and CUB2 appears to be appreciably smaller than observed for ADAMTS13 CUB1-2. The extensive interdomain cleft appears to be highly important for ADAMTS13 CUB1-2, as several of the surface features, including pockets, P1 and P2, are formed by this interface and involve amino acids from both domains (Fig. 2). Notably, amino acids at the CUB1-CUB2 interface (Leu^1221^, Arg^1247^, Asn^1267^, Val^1270^, Gln^1302^, Phe^1304^, Gly^1305^, Phe^1328^, Asn^1330^, and Pro^1333^) are particularly highly conserved between species, reflecting their importance in the overall fold of the tandem CUB1-2 domains (fig. S6). Ca^2+^-binding CUB domains often interact with their ligands through a conserved mechanism that relies upon the Ca^2+^-binding site in the odd numbered loops (Fig. 3) (*31*). The lack of Ca^2+^ binding in ADAMTS13 CUB1-2 and the presence of N-linked glycans on loops L5 and L13 in CUB1 and CUB2, respectively, strongly support the contention that these domains interact with their ligand differently.

In loop L9 in CUB1, the unpaired Cys^1275^ (that we substituted to Ser) is not present in other CUB domains. Previous studies have reported the ability of Cys^1275^ to undergo redox reactions that may regulate VWF function (*40*–*42*). We certainly found that, without the C1275S mutation, there appeared to be some dimerization of the recombinant CUB1-2 domains in S2 cells. However, it should be recognized that the location of this free Cys, and the presence of the N-linked glycan on the adjacent L5 loop, does mean that Cys^1275^ would not ordinarily be accessible/available for redox reactions. It is also worth highlighting that Cys^1275^ is only found in humans and chimpanzees, which therefore does not support an important or conserved role in the regulation of VWF function (fig. S6).

To delineate how the ADAMTS13 CUB1-2 domains interact with the central Spacer domain, we performed unbiased docking simulations using HADDOCK software. Highly encouragingly, this approach provided several poses that favored the previously described region of the Spacer domain implicated in CUB domain binding (fig. S3) (*7*, *19*). From these simulations, we identified a panel of surface-exposed amino acids on CUB1-2 that might contribute to Spacer domain binding (fig. S4). To screen these amino acids for their contribution to Spacer domain binding, we introduced substitutions into full-length ADAMTS13. Functional analysis of these variants in the absence and presence of the activating anti-CUB antibody 17G2 (specifically enhances ADAMTS13 function by disruption of the Spacer-CUB binding) identified those ADAMTS13 variants the Spacer-CUB interaction had been perturbed (*7*, *8*, *15*). This approach is perhaps the most sensitive and meaningful way to ascribe functional importance to each residue, as it assays the effect of the substitutions in the full-length, native protein. We identified six amino acids (Trp^1245^, Trp^1250^, and Lys^1252^ in CUB1 and Arg^1326^, Glu^1387^, and Glu^1389^ in CUB2), which, when mutated, resulted in the near-complete disruption of Spacer-CUB binding (Fig. 4, A to Q). These residues form two clusters of amino acids located on the same face of the tandem CUB1-2 domains (Fig. 4S). Amino acid substitutions that had an intermediate effect on the Spacer-CUB interaction (Lys^1265^, Arg^1361^, and Glu^1382^) are in proximity to these two clusters. A fortuitous control for this assay was provided by one of the ADAMTS13 variants (R1219Q) that resulted in loss of binding of the 17G2 antibody and was therefore not activated, demonstrating the specificity of the assay for measuring disruption of the Spacer-CUB interaction (Fig. 4R). It seems unlikely that Arg^1219^ plays a direct role in the Spacer interaction given its location on the opposite side of the CUB domains to the proposed interaction site.

Given the evidence for involvement of these two clusters of amino acids in interacting with the Spacer domain, we performed further analyses using HADDOCK and ClusPro to generate a refined docking model. We selected the highest-ranking docking pose (Fig. 5), which agreed very well with the functional data (Fig. 4). That the highest scoring poses from both docking programs were very similar provides compelling evidence that they closely approximate to the Spacer-CUB interaction. We propose an interaction that, in addition to the residues identified functionally, also includes contributions of Leu^1248^ and Arg^1272^ in CUB1 and Leu^1366^ in CUB2. Further confidence in this interaction site is provided by the excellent complementarity with the exosite on the surface of the Spacer domain (Fig. 5). Moreover, inspection of the conservation of these amino acids between species reveals that all of the residues in the CUB2 domain site (Arg^1326^, Arg^1361^, Glu^1387^, and Glu^1389^) predicted to form direct interactions with the Spacer are perfectly conserved from humans to fish, which is a strong indication of functional importance (fig. S6, A and B). Our docked model reveals that this CUB2 site corresponds to the region that accommodates the well-described L9 Spacer domain loop containing Arg^660^, Tyr^661^, and Tyr^665^ that has been previously implicated in CUB domain binding (*7*, *19*). Notably, the reciprocal Spacer domain site residues Tyr^661^ and Tyr^665^ are also perfectly conserved between species, and the presence of either Arg or Lys at position 660 corroborates the contention that the CUB-Spacer interaction is evolutionarily conserved (fig. S7). The conservation of the CUB1 site is also high—more so for the charged residues Lys^1252^ and Arg^1272^, but the hydrophobicity of Trp^1245^, Leu^1248^, and Trp^1250^ is generally well conserved (fig. S6, A and B), as are the reciprocal hydrophobic residues in the Spacer domain Leu^591^, Phe^592^, and Leu^637^ (fig. S7). Spacer-CUB–mediated global latency in ADAMTS13 is found in mammals, birds, reptiles, amphibians, and fish, despite appreciable variability in the number of intervening TSP repeats between the Spacer and CUB domains (*10*). The ADAMTS13 Spacer-CUB interaction can also be perturbed by reducing the pH to 6, which, in turn, enhances proteolysis of short VWF A2 domain fragment substrates (*8*, *10*, *43*). As the maximal enhancing effect is detected at pH 6, the protonation of His residues in ADAMTS13 is likely responsible for the disruption of the Spacer-CUB interaction. Although His^1364^ is at the center of the interaction site in human ADAMTS13, this residue is not well conserved in other species that are also enhanced by pH 6 (fig. S6). This may therefore suggest that the enhanced enzymatic function of ADAMTS13 at pH 6 is not manifest by directly influencing Spacer-CUB binding site residues, but, rather in an indirect manner, through protonation of His residues throughout ADAMTS13 that, together, alter ADAMTS13 conformation.

Some studies have suggested that the N-linked glycans in the ADAMTS13 CUB domains may directly influence the Spacer-CUB interaction (*44*). Given their radial positions on the CUB1 and CUB2 domains, however, they are not well situated to contribute directly. Moreover, the variable conservation of the N-linked glycans does not perhaps support a direct role for these in mediating the Spacer-CUB interaction. However, removal of the glycans may have the potential to alter the overall conformation of the CUB domains that disrupts the Spacer-CUB interaction. The glycosylation site on Asn^1235^ in CUB1 is quite well conserved between species (fig. S6) but is notably absent in mice, rats, canines, elephants, and some fish. An additional glycosylation consensus sequence is also found in loop L2 of CUB1 in some birds, reptiles, and amphibians, although it is unclear whether this site is occupied in these species (fig. S6). The glycosylation site on Asn^1354^ in CUB2 is poorly conserved between species, where it is either absent or potentially located in different positions on loop L13.

Previous studies have analyzed the globally latent conformation of full-length ADAMTS13 through small-angle x-ray scattering and electron microscopy (*7*–*10*). However, these studies are unable to ascertain how the Spacer-CUB interaction is manifest or how the Spacer-CUB interaction might be broken/disturbed. This latter point is of particular interest given that modulation of the Spacer-CUB interaction reversibly controls ADAMTS13 global latency. Binding of either VWF, certain anti-MP, anti-Spacer, or anti-CUB monoclonal antibodies to ADAMTS13 can induce loss of global latency through perturbation of Spacer-CUB binding (*7*, *8*, *45*). This implies that this interaction is conformationally sensitive. Global latency is likely controlled by structural changes induced by ligand binding. Such changes likely alter the orientations of critical amino acid at the Spacer-CUB interface. Subtle changes that disrupt this interaction, in turn, induce larger conformational changes in the rest of molecule. It is interesting to note that many anti-ADAMTS13 autoantibodies from TTP patients recognize cryptic epitopes in ADAMTS13 that are only exposed when the Spacer-CUB interaction is disrupted. Moreover, several autoantibodies from TTP patients that induce opening of ADAMTS13 have also been described, which would consequently be predicted to augment further immune complex formation. Together, these findings reveal a potentially important contribution of ADAMTS13 conformation in autoimmune recognition of ADAMTS13 (*20*, *46*).

Proteases are classically produced in latent forms without enzymatic activity. Specific regulation of enzyme latency and activity is central to the spatial and temporal control of protease function. Latency can be conferred through multiple mechanisms. Conversion of a latent enzyme to an active form frequently relies upon proteolytic activation, enzyme allostery, and/or activating cofactors. ADAMTS13 is secreted with its prodomain removed. Therefore, activation of ADAMTS13 does not rely upon specific, on-demand proteolytic activation, like other hemostatic proteases. Instead, ADAMTS13 proteolytic function is controlled allosterically by its substrate, VWF (*13*). Control of ADAMTS13 latency is complex involving two distinct latency mechanisms that we term global latency and local latency. Global latency conferred by the Spacer-CUB interaction maintains ADAMTS13 in a closed, compact conformation in circulation. The Spacer-CUB interaction also influences that the conformation of the MP domain, which we propose, consolidates the local latency mechanism. Local latency is conferred by the ionic interactions of the gatekeeper triad residues in the active site cleft (*13*). Together, these latency mechanisms prevent nonspecific proteolysis by ADAMTS13 while in circulation. Conversion of ADAMTS13 into an active protease only requires VWF to first unravel. The interaction of the ADAMTS13 CUB domains is often considered the first event in recognition of VWF. This occurs through binding to the D4 domain (*8*). In binding the CUB domains, we propose that the D4 domain induces a structural shift in the CUB interface that induces their dissociation from the Spacer domain. This not only opens ADAMTS13 but also transduces some structural changes to the MP domain that perhaps improves the efficiency of release of local latency. Interaction of the ADAMTS13 MDTCS domains with the unraveled VWF A2 domain provides much of the high-affinity binding between enzyme and substrate. The Dis domain interaction with VWF is of particular importance as it is this that allosterically activates the MP domain to facilitate substrate proteolysis (*13*). After the proteolytic event, rheological shear forces no longer influence the cleaved A2 domain, which likely promotes dissociation of ADAMTS13 from VWF. In so doing, ADAMTS13 recycles by reverting to its globally latent and locally latent conformation.

In this study, we report the first crystal structure of any C-terminal domains of an ADAMTS family member. We reveal a novel configuration of the ADAMTS13 tandem CUB1-2 domains manifest by the more extensive interface between these domains that contributes to the formation of two surface pockets. Although the ADAMTS13 CUB1-2 domains bear close structural resemblance in their organization of their β strands to other tandem Ca^2+^-binding CUB domains, these domains do not coordinate Ca^2+^, nor do they use their radial, odd numbered loops for ligand binding. Rather, the ADAMTS13 CUB1-2 domains have two highly conserved surface patches that form an extended binding site for the central ADAMTS13 Spacer domain. The interaction of the CUB1-2 domains with the Spacer domain controls ADAMTS13 global latency by locking ADAMTS13 in a closed conformation that is only released, physiologically, when ADAMTS13 interacts with VWF.

## MATERIALS AND METHODS

### Expression and purification of MBP-CUB1-2-His C1275S

The complementary DNA encoding the ADAMTS13 CUB1-2 domains (S1189-T1427) was cloned into the insect cell expression vector pMT-PURO-MBP. Primers used for amplification and cloning were CGCGCAGACTAATGCGGCCGCATCCAGTGCCTGTGGCAGG and TGGTGATGATGACCGGTACGCGTGGTTCCTTCCTTTCCCTTCCAG. ADAMTS13 CUB1-2 was cloned in frame with an N-terminal MBP tag and a 6xHis tag at the C terminus for purification. The surface-exposed unpaired cysteine, Cys^1275^, was mutated to Ser (C1275S) by site-directed mutagenesis and verified by sequencing to prevent intermolecular disulfide bond formation. Primers used for site-directed mutagenesis were GTGGTGAGGCAGCGCTCCGGGCGGCCAGGAGGT and ACCTCCTGGCCGCCCGGAGCGCTGCCTCACCAC.

This vector was transfected into Drosophila S2 cells using calcium phosphate, and stably transfected cells were selected using Schneider’s Drosophila medium (Lonza) containing 10% (v/v) fetal calf serum and puromycin (10 μg ml^−1^) (Gibco) for 3 to 4 weeks. Cells stably expressing MBP-CUB1-2-His (C1275S) were expanded to 1 liter in EX-CELL 420 serum-free medium (Sigma-Aldrich)/puromycin (10 μg ml^−1^) in 2.5-liter flasks at 27°C/120 rpm to a cell density of 2 × 10^6^ to 4 × 10^6^ cells ml^−1^. To these, copper sulfate (0.5 mM final concentration) was added to induce protein expression. Cells were cultured for a further 7 days, and media were harvested by centrifugation and filtration. Conditioned media were concentrated by tangential flow filtration using a 10-kDa filtration unit (Millipore) and dialyzed into 20 mM Hepes (pH 7.5), 0.5 M NaCl, 5 mM CaCl_2_, and 5 mM maltose. The dialyzed medium was applied to a HiTrap Chelating HP column (GE Healthcare) bound with Ni^2+^. The column was washed, and MBP-CUB1-2-His (C1275S) was eluted by stepwise increases in imidazole concentration (30, 60, 100, 150, 200, 250, and 300 mM). Fractions were analyzed by SDS–polyacrylamide gel electrophoresis and Coomassie staining, and the 100 mM imidazole elution fractions containing the highest concentrations of MBP-CUB1-2-His (C1275S) were dialyzed into 20 mM Hepes (pH 7.5), 150 mM NaCl, 5 mM CaCl_2_, and 5 mM maltose and further purified by gel filtration using a HiPrep 26/60 Sephacryl S-200HR column (GE Healthcare). The peaks containing monodisperse MBP-CUB1-2-His (C1275S) were concentrated using Amicon centrifugal filter (10-kDa cutoff) to 4 to 7 mg ml^−1^.

### Crystallization of MBP-CUB1-2-His (C1275S) and x-ray data collection

Crystals of MBP-CUB1-2-His (C1275S) were grown using hanging-drop vapor diffusion at 20°C in 24-well VDX plates (Hampton Research). Initial crystallization conditions were established with screening kits from Hampton Research (Index) and from Molecular Dimensions (JCSG Plus, MIDAS, ProPlex, and Structure Screen I and II) using sitting-drop vapor diffusion with mosquito crystallization robotics (SPT Labtech, UK). For the optimal growth of MBP-CUB1-2-His (C1275S) crystals, each drop was prepared by mixing 1 μl of MBP-CUB1-2-His (C1275S) (5 mg ml^−1^) and 1 μl of precipitant solution [21% (v/v) PEG1500 (polyethylene glycol, molecular weight 1500), 100 mM sodium propionate, sodium cacodylate trihydrate, 200 mM MnCl_2_, and bis-tris propane (pH 5)]. For crystal freezing, the crystals were flash-frozen in a stream of nitrogen gas at 100 K. Diffraction data were collected on an Eiger2 X16M detector system at the I04 experimental station of the Diamond Light Source, UK. Data were processed and scaled using the autoPROC program. Further data analysis was carried out using CCP4 suite. The crystals belonged to space group *P*3_2_21 and contained one molecule per asymmetric unit. Data collection statistics are summarized in Table 1.

### MBP-CUB1-2-His (C1275S) structure determination and refinement

To determine the structure of MBP fusion CUB1-2 domains, we used molecular replacement with Phaser (CCP4 suite) using the structure of MBP as a search model (PDB code: 3O3U). Clear electron density for β-strand CUB motifs was visible in the 2*F*_o_*-F*_c_ map. Refinement of the crystal structure was achieved through iterative cycles of model building using COOT, followed by refinement of the models with Refmac5 and Phenix. A 5% portion of the data was set aside before the refinement for the *R*_free_ calculations for dataset. Solvent molecules became apparent in the later stages of refinement. Refinement was pursued until no further decrease in *R*_free_ was observed. Refinement statistics are summarized in Table 1. Structural alignments were carried out using PyMOL (http://www.pymol.org), which was used for the generation of all figures.

### Docking simulations of ADAMTS13 CUB1-2 domains with ADAMTS13 Spacer domain

Three-dimensional models of the ADAMTS13 CUB1-2 domain complex with the ADAMTS13 Spacer domain were calculated using separate docking programs ClusPro 2.0 and HADDOCK 2.2 (*47*, *48*). Crystal structures are available for the ADAMTS13 Spacer domain from the MDTCS structure at 2.8-Å resolution (PDB code: 6QIG) and three DTCS structures (PDB codes: 3GHM, 3GHN, and 3VN4 at resolutions 2.6, 2.8, and 2.8 Å, respectively). We recently improved the MDTCS structure resolution to 2.2 Å (unpublished), which is the highest resolution structure available for the ADAMTS13 Spacer domain. The MBP residues were removed from the CUB1-2 structure. Docking calculations were first performed with ClusPro without any assumptions as to the residues involved in the interface from either the Spacer or CUB1-2 structure. The Spacer domain was defined as the receptor and CUB1-2 as the ligand. ClusPro performed 70,000 rotations of the ligand and for each rotation translation relative to the receptor on a grid. The translations with the best score from each rotation were selected from the 1000 rotation/translation combinations that have the lowest score (sampling from around 10^9^ positions). Using all the available templates for the Spacer, a variety of docking poses resulted in diverse combinations of the surfaces from the CUB1-2 domains and the Spacer domain. Many orientations could be ruled out based on the stereochemical position of the N and C termini. Only one orientation placed CUB1-2 consistently in the top ten scoring poses for all calculations, and this occurred in the top 10 p-scoring poses for all calculations. Docking simulations were also calculated with HADDOCK using a different approach by specifying residues in the Spacer domain for which experimental data had implicated involvement in the CUB interaction—i.e., Arg^660^, Tyr^661^, and Tyr^665^. These were defined as active residues. As no experimental data on interacting residues from the CUB1-2 structure were available, all surface-accessible residues were allowed to participate in docking.

A final phase of the docking was performed specifying involvement of residues Trp^1245^, Trp^1250^, and Lys^1252^ in CUB1 and Arg^1326^, Glu^1387^, and Glu^1389^ in CUB2 and the Spacer domain Arg^568^, Phe^592^, Arg^660^, Tyr^661^, Glu^664^, and Tyr^665^. This resulted in two poses that both oriented the Spacer domain loops to pockets P1 and P2, but the HADDOCK prediction shifted translationally toward the P2 pocket such that differences in the contacts appear at the periphery of the interaction. Thus, both docking simulations predict the salt bridge between Glu^634^ and Lys^1252^ in the P1 pocket, but due to the shift toward P2 in the HADDOCK simulation, there is a loss of the Asp^635^ interaction with P1 Arg^1272^ and instead interactions are gained with the P2 pocket such as Glu^664^ -Arg^1297^ and Leu^668^-Phe^1295^, which do not occur in the ClusPro simulation.

### Generation of full-length ADAMTS13 CUB domain mutants

The wild-type human ADAMTS13 mammalian expression vector (pcDNA3.1) with a 6xHis tag fused to the C terminus has been described previously. DNA fragments containing single point and composite CUB1-2 domain mutations (R1219Q, W1245A + W1250A, R1251Q + K1256Q, K1252Q, K1265Q, F1295A + Y1296D, R1297Q, W1307A + W1406A, E1309Q, R1326Q, H1334Q, R1336Q, R1361Q, E1382Q, E1387Q, E1389Q, and E1392Q) were synthesized (Thermo Fisher Scientific) and subcloned into the wild-type ADAMTS13 vector using Mre I and Xba I restriction enzymes. Vectors were verified by sequencing. ADAMTS13 and ADAMTS13 mutants were transiently transfected into HEK293T cells using Lipofectamine and expressed in Opti-MEM (Invitrogen). After 3 days, conditioned media were harvested and centrifuged. Expression and secretion were analyzed by Western blotting using an anti-6xHis antibody. ADAMTS13 concentrations were measured by ELISA.

For kinetic analyses, wild-type and variant ADAMTS13 in conditioned medium and VWF96 were incubated separately in 20 mM tris (pH 7.8), 150 mM NaCl, 5 mM CaCl_2_ (TBSC), and 1% bovine serum albumin (BSA) at 37°C for 15 min. Reactions were set up containing 0.45 to 0.6 nM ADAMTS13, or ADAMTS13 mutant, and 500 nM VWF96 in TBSC/1% BSA in the presence and absence of the activating anti–CUB1-2 monoclonal antibody 17G2 (10 μg ml^−1^). Ten microliters of reaction subsamples was stopped between 0 and 90 min with EDTA. The stopped subsamples were diluted to 0.75 nM VWF96 in TBS/1% BSA buffer and analyzed by VWF96 ELISA to quantify the concentration of uncleaved substrate. For this, anti-His G antibody (1.08 μg ml^−1^) (Invitrogen) was adsorbed onto 96-well microtiter plates in 50 mM sodium carbonate/bicarbonate (pH 9.6) overnight at 4°C. Wells were washed with TBS/0.1% Tween and blocked with TBS/3% BSA for 2 hours. Wells were washed, and a standard curve of purified full-length VWF96 (0 to 1.2 nM) diluted in TBS/1% BSA was added. In parallel, stopped samples were incubated at room temperature for 1 hour. After washing, peroxidase-conjugated anti–herpes simplex virus immunoglobulin G (0.5 μg ml^−1^) (Bethyl) diluted in TBS/1% BSA was added and incubated for 1 hour. Wells were washed, and 170 μl of SIGMAFAST *o*-phenylenediamine dihydrochloride peroxidase substrate (Sigma-Aldrich) was used for the detection. Color development was stopped using 2.5 M H_2_SO_4_ and measured spectrophotometrically at 492 nm.

The concentration of uncleaved VWF96 at each time point was measured from the standard curve, and from this, the fraction of substrate proteolyzed was calculated and plotted as a function of time. For each ADAMTS13 variant, the number of reactions performed is provided in the figure legends. The fold activation induced by incubation of the 17G2 antibody was determined using GraphPad software (Prism) by fitting the data from the time-course reactions into the equation, *P* = 1 − exp(−1 × [ADAMTS13] × *t* × *k*_cat_/*K*_m_), where *P* is the fraction of VWF96 proteolyzed and *t* is the time in seconds. From this, the percentage change in catalytic efficiency, *k*_cat_/*K*_m_, in the presence of 17G2 was derived. Differences in percentage activation induced by 17G2 between wild-type ADAMTS13 and ADAMTS13 mutants were analyzed by one-way analysis of variance (ANOVA).

## SUPPLEMENTARY MATERIALS

Supplementary material for this article is available at http://advances.sciencemag.org/cgi/content/full/7/16/eabg4403/DC1

## Appendix

View/request a protocol for this paper from *Bio-protocol*.
